# Development of a PATIENT-Medication Adherence Instrument (P-MAI) and a HEALTHCARE PROFESSIONAL-Medication Adherence Instrument (H-MAI) using the nominal group technique

**DOI:** 10.1371/journal.pone.0242051

**Published:** 2020-11-11

**Authors:** Sheron Sir Loon Goh, Pauline Siew Mei Lai, Su-May Liew, Kit Mun Tan, Wen Wei Chung, Siew Siang Chua

**Affiliations:** 1 Department of Primary Care Medicine, Faculty of Medicine, University of Malaya, Kuala Lumpur, Malaysia; 2 Department of Medicine, Faculty of Medicine, University of Malaya, Kuala Lumpur, Malaysia; 3 Department of Pharmacy, University Malaya Medical Centre, Kuala Lumpur, Malaysia; 4 School of Pharmacy, Faculty of Health and Medical Sciences, Taylor’s University, Subang Jaya, Selangor, Malaysia; The University of Sydney School of Pharmacy, AUSTRALIA

## Abstract

To date, several medication adherence instruments have been developed and validated worldwide. However, most instruments have only assessed medication adherence from the patient’s perspective. The aim was to develop and validate the PATIENT-Medication Adherence Instrument (P-MAI) and the HEALTHCARE PROFESSIONAL-Medication Adherence Instrument (H-MAI) to assess medication adherence from the patient’s and healthcare professional (HCP)’s perspectives. The P-MAI-12 and H-MAI-12 were developed using the nominal group technique. The face and content validity was determined by an expert panel and piloted. The initial version of these instruments consisted of 12 items were validated from October-December 2018 at a primary care clinic in Malaysia. Included were patients aged ≥21 years, diagnosed with diabetes mellitus, taking at least one oral hypoglycaemic agent and who could understand English. The HCPs recruited were family medicine specialists or trainees. To assess validity, exploratory factor analysis (EFA) and concurrent validity were performed; internal consistency and test-retest were performed to assess its reliability. A total of 120/158 patients (response rate = 75.9%) and 30/33 HCPs (response rate = 90.9%) agreed to participate. EFA found three problematic items in both instruments, which was then removed. The final version of the P-MAI-9 and the HMAI-9 had 9 items each with two domains (adherence = 2 items and knowledge/belief = 7 items). For concurrent validity, the total score of the P-MAI-9 and the H-MAI-9 were not significantly different (p = 0.091), indicating that medication adherence assessed from both the patient’s and HCP’s perspectives were similar. Both instruments achieved acceptable internal consistency (Cronbach’s α: P-MAI-9 = 0.722; H-MAI-9 = 0.895). For the P-MAI-9, 7/9 items showed no significant difference between test and retest whereas 8/9 items in the H-MAI-9 showed significant difference at test and retest (p>0.05). In conclusion, the P-MAI-9 and H-MAI-9 had low sensitivity and high specificity suggesting that both instruments can be used for identifying patients more likely to be non-adherent to their medications.

## Introduction

Medications are frequently used in treating chronic conditions and extend life expectancy [1]. However, previous studies reported that only 50% of patients adhered to their medications [2, 3]. This is a major concern as low adherence causes medication wastage, morbidity and mortality [4]. In 2012, medication adherence was redefined as “the process by which patients take their medications as prescribed” [5], and consists of three main components: initiation (when the first dose of prescribed medication has been taken), implementation (when a patient’s actual dosing corresponds to the prescribed dosing regimen from initiation until the last dose is taken) and discontinuation (end of therapy) [5].

To date, there is no gold standard on how medication adherence should be assessed. Pharmacy refilling data shows when patients refill their medications but does not report adherence in real-time [6]. The electronic pillbox provides continuous data for the monitoring of medication adherence [7] as it reports the opening time of the pillbox. However, this method is expensive [8] and is unable to report actual adherence as patients may open the container and not take their medications [7]. Self-reported medication adherence instruments assess medication adherence subjectively, as it is based on patient’s recall [9]. These instruments are easy to administer in routine clinical practice as patients take less than 10 minutes to answer these questionnaires [10]. However, the most common drawback is that patients tend to overestimate their medication adherence to avoid disapproval from healthcare professionals (HCPs) [10].

Many self-reported medication adherence instruments have been developed and validated worldwide [11] [S1 Table]. Thirty instruments were validated in America [12–40], 13 in Europe [10, 41–51], three in Asia [52–54], two in Australia [55, 56] and one in Africa [57]. The number of items for these instruments ranged from 1–30 items. Three studies performed both EFA and CFA [35, 45, 53], thirteen studies reported only performed EFA [20, 21, 26, 34, 36–39, 50, 54–56, 58] whilst two studies performed only CFA [29, 30]. Twelve studies assessed both internal consistency and test-retest [13, 20, 30, 34, 37, 39, 50, 52, 54–56, 58], 17 studies assessed only internal consistency [10, 21, 23, 26–29, 32, 33, 35, 36, 40, 43, 45, 47, 53, 57] whilst two studies only assessed stability [31, 38]. All 48 studies only assessed medication adherence from the patient’s perspective [10, 12–27, 29–57] except one study which only assessed from the HCP’s perspective [28]. Previous literature has recommended that adherence to medications should be assessed by more than one method as it can offer more accurate and reliable information [59]. It is well known that patients tend to overestimate their adherence to medications when using self-reported medication adherence instruments for fear of reprimand from their HCP [60]. Additionally, a doctor can also assess a patient’s medication-taking behaviour during a doctor-patient consultation through direct observation, measurement of laboratory parameters, patient’s lifestyle, values and preferences for care [61]. However, some evidence found that doctors may be poor judges in assessing their patient’s medication adherence [62, 63] as they may not be aware when their patients discontinue their medication [28]. To date, no instrument has been developed and validated specifically from two different perspectives. Therefore, this study aimed to develop and validate two self-reported medication adherence instruments: 1) The Patient-Medication Adherence Instrument (P-MAI) to assess medication adherence from the patients’ perspective and 2) the Healthcare Professional-Medication Adherence Instrument (H-MAI)] to assess patient’s medication adherence from the HCP’s perspective.

## Materials and methods

The study was divided into two parts: the development of the P-MAI and H-MAI and its validation. Mirzaei et. al., 2019 [64] and Grew et. al., 2019 [65] which described the development and validation of a questionnaire of service quality in community pharmacy; and El-Den et. al., 2020 [66] which described the psychometric principles for the development and validation of measurement instruments have been used as references to guide this work.

### Part 1: Development of the Patient-Medication Adherence Instrument (P-MAI) and the Healthcare Professional-Medication Adherence Instrument (H-MAI)

The P-MAI and H-MAI were developed from May to September 2018.

### Item generation

All items that were considered in the development of the P-MAI and the H-MAI were obtained from literature search. A list of 40 potential items (P-MAI = 20 items; H-MAI = 20 items) was identified from the 49 validated self-reported instruments reported in S1 Table [10, 12–57]. Then, these potential items were presented to the members of the NGT as a starting point for them to develop the items of both P-MAI and H-MAI.

The nominal group technique (NGT) was then used to develop the content of the two instruments. NGT is a highly structured face-to-face group interaction, which provides participants with an equal opportunity to have their opinions heard and considered by other group members [67]. Compared to the Delphi method, the NGT is a faster method to generate ideas for developing instruments as it can be used to explore ideas of different health care professionals and laypeople in one session [67]. We decided to develop the P-MAI and H-MAI in English as it is understood by most Malaysians and HCPs [68].

Twenty persons were approached to participate in the NGT, but only seven persons (two patients with a chronic disease, two doctors, two pharmacists and one nurse) agreed to participate. The NGT was conducted in five stages: introduction, silent generation, round-robin, clarification and ranking. Firstly, participants were given a brief introduction and explanation of the purpose and procedure of the meeting. During the silent generation, each participant was provided with a list of items that may be included in the P-MAI. They were required to read and understand the items carefully. No discussion was allowed at this stage. During the round-robin process, participants were given the opportunity, one at a time, to share their ideas, which were recorded on a flip chart. The round-robin continued until all ideas were presented. No debate or discussion of ideas occurred at this stage. Then, clarification of ideas was generated through discussion. At this stage the wording of any unclear ideas/items was clarified, similar ideas/items were combined to form hybrid ideas/items, and new ideas/items were allowed to be presented, but no ideas/items were eliminated. Each participant was then asked to rank the importance of ideas based on a 10-point Likert scale (1 = least important; 10 = most important) independently. The entire process was repeated for the H-MAI. At the end of the NGT, the final draft of the P-MAI-20 had three domains (adherence, knowledge and belief) with 20 items, whilst the H-MAI-17 had three similar domains with 17 items.

### Face and content validity

The P-MAI-20 and the H-MAI-17 were then reviewed by an expert panel (which consisted of one family medicine specialist, one geriatrician, two academic pharmacists and two clinical pharmacists). The expert panel decided to reduce the P-MAI from 20 items to 12 items and the H-MAI from 17 to 12 items as some items were found to be too lengthy or did not fit into the domains. Responses for both instruments were measured on a 5-point Likert scale ranging from strongly disagree = 1 to strongly agree = 5. A higher score indicates better adherence to medications.

### Pilot test

We then piloted the P-MAI-12 in two patients with diabetes mellitus (DM) who could understand English whilst the H-MAI-12 was piloted by the patient’s doctor during their doctor-patient consultation. No problems were encountered. Therefore, no further modifications were made to the P-MAI-12 and H-MAI-12.

### Part 2: Validation of the Patient-Medication Adherence Instrument (P-MAI) and Healthcare Professional-Medication Adherence Instrument (H-MAI)

### Study design

The P-MAI-12 and H-MAI-12 were validated from October to December 2018, in an urban tertiary-based primary care clinic in Malaysia.

### Participants

Included were patients aged ≥21 years, diagnosed with DM, taking at least one oral hypoglycaemic agent and could understand English. We included individuals who were diagnosed with diabetes mellitus (DM) as self-management (including adherence to medications) plays a crucial role in the control of this chronic health problem [69]. In addition, all patients with DM have their HbA1c assessed one week prior to their clinic appointment. Therefore, HbA1c values can be used as an objective measurement [70] to correlate with a patient’s medication adherence. Patients who had intellectual disabilities were excluded. The HCPs recruited were family medicine specialists and trainees working in an urban tertiary-based primary care clinic in Malaysia.

### Sample size

The sample size was calculated based on the number of items to participants ratio of 1:10 to perform factor analysis [71]. There are 12 items in the P-MAI-12 and H-MAI-12, respectively. Therefore, the total number of participants required were 120 patients for P-MAI-12 and 120 H-MAI-12 assessments by HCPs.

### Instruments used

#### Baseline demographic questionnaire

A baseline demographic questionnaire was used to collect patient’s socio-demographic data, medical history, medication adherence status and their reasons for non-adherence. Another baseline demographic questionnaire was used to collect their HCP’s socio-demographic data and of the length of working experience as a doctor.

#### Patient-Medication Adherence Instrument (P-MAI-12)

This instrument consists of 12 items with 3 domains: adherence, knowledge and belief. Patients were asked to fill the P-MAI-12 based on their recall of how they took their medications. All responses were measured on a 5-point Likert scale, ranging from strongly disagree = 1 to strongly agree = 5. A higher score indicates better medication adherence.

#### Healthcare Professional-Medication Adherence Instrument (H-MAI-12)

This instrument mirrored the P-MAI-12; it has 12 items with 3 domains: adherence, knowledge and belief. The patient’s HCP was asked to fill the H-MAI-12 based on how they perceived their patients’ medication-taking behaviour. All responses were measured on a 5-point Likert scale, ranging from strongly disagree = 1 to strongly agree = 5. Similarly, a higher score implies better medication adherence.

### Data collection

#### Patients

Potential patients were recruited using a 1:10 systematic random sampling approach. Each day, a number from 1–10 was randomly selected using a computerized random number generator in Microsoft Office^®^ Excel^®^ (Washington, United States of America). Based on the random number produced, the first potential patient was approached at the triage counter. The purpose of the study was explained using the participant information sheet. For those who agreed to participate, written informed consent was obtained. Patients were then asked to fill the baseline demographic form and the P-MAI-12 while they were waiting to see their HCPs. This took approximately 5–10 minutes. The completed instruments were checked by the researcher to ensure that all items were answered. Two weeks later, the P-MAI-12 was re-administered by the researcher to the same patients over the telephone.

#### Health care professionals

The researcher approached each HCP individually and explained the purpose of the study using the participant information sheet. For those who agreed to participate, written informed consent was obtained. HCPs were asked to complete the baseline demographic form, followed by the H-MAI-12 based on how they perceived their patients were taking their medications during/immediately after their doctor-patient consultation. Two weeks later, the H-MAI-12 was completed by the same HCPs after a doctor-patient telephone consultation.

### Ethics approval

Ethics approval was obtained from the Medical Research Ethics Committee of University Malaya Medical Centre (MREC ID NO: 2018326–6167) before the commencement of the study.

### Data analysis

Data were analysed using Statistical Package for the Social Sciences (SPSS) version 21 (Illinois, United States of America). Normality was assessed using the Kolmogorov–Smirnov test. As data were not normally distributed, continuous variables were expressed as median and interquartile range, whilst categorical data were presented as frequency and percentage. A p-value of <0.05 was considered statistically significant.

#### Validity

*Exploratory factor analysis*. Exploratory factor analysis (EFA) was used to explore the dimensions of both instruments. The Keiser-Meir-Olkin (KMO) test (> 0.7), the Bartlett test of sphericity, anti-image correlation matrix coefficients (> 0.5), factor loadings (> 0.4) and communality were measured. Items that had low communalities (< 0.3) were removed and EFA was re-analysed. The normality of data was assessed using the skewness, kurtosis and Kolmogorov–Smirnov test. The skewness (<-1.0), kurtosis (>1.0) and Kolmogorov–Smirnov test (p<0.001) indicates that data is not normally distributed. Hence, the principal axis factor was used as the data has violated the assumption of multivariate normality [72]. As items in the instruments could be inter-related, the promax (oblique) rotation method was used [72].

*Concurrent validity*. The Mann Whitney U test was used to compare the differences of total score between the P-MAI and H-MAI.

*Flesch reading ease*. Flesch reading ease is a tool for calculating the approximate reading level of English-language content that was used to determine the readability of both instruments. This was calculated using Microsoft Office^®^ Word^®^ 2016 (Microsoft Corporation, Redmond, Washington, United States). The calculation was based on the average number of syllables per word and words per sentence. The higher the Flesch score, the easier it is to understand the document. A Flesch reading ease score between 60 to 70, indicates the document is easily understood by 13-15-year-old students. A score between 70 to 80, suggests that the document is fairly easy to read [73].

#### Reliability

Cronbach’s alpha was used to calculate the internal consistency of both instruments. A Cronbach’s alpha values more than 0.9 indicates redundancy of some items, values 0.70–0.90 suggest adequate internal consistency, values 0.60–0.70 imply acceptable internal consistency and values below 0.50 indicates unacceptable internal consistency [74]. The corrected item-total correlation was also performed where corrected item-total values are considered as acceptable when the values are >0.2. The Wilcoxon signed-rank test was used to assess the reliability of the instruments at test and retest to identify any items found ambiguous, and could not be interpreted in the same manner over time [75].

The total score of the P-MAI and the H-MAI is the sum of all items (score range: 9–45). A higher score indicates better medication adherence. Non-adherence was defined when the total score ranged from 9 to 35 (calculated total score<80%); whilst adherence was defined as a total score, which ranged from 36 to 45 (calculated total score ≥80%).

#### Sensitivity and specificity

The sensitivity, specificity, and predictive values were calculated. The sensitivity of both instruments determines the ability to correctly predict good glycaemic control (HbA1c≤7%) in patients who were adherent to their medications while the specificity of the instruments determines the ability to correctly predict poor glycaemic control (HbA1c>7%) in patients who were non-adherent to their medications [76, 77]. We selected the level of HbA1c ≤7% as good glycaemic control as the median age of the participants in our study was 63 years of age. A lower HbA1c target (≤6.5%) is usually used in newly diagnosed diabetes patients, younger age, healthier and low risk of hypoglycaemia [76]. Positive predictive value predicts the likelihood of patient adherence to his/her medications when assessed using both P-MAI and H-MAI, which is associated with good glycaemic control, while negative predictive value predicts the likelihood of patient non-adherence as assessed using both instruments, which is associated with poor glycaemic control [77].

## Results

A total of 120/158 patients (response rate = 75.9%) and 30/33 HCPs (response rate = 90.9%) agreed to participate in this study. In a normal clinic setting, each HCP will see more than one patient per day. Hence, these 30 HCPs were recruited to perform 120 HMAI-12 assessments for 120 patients. The median age of patients and their HCPs were 63 (57–69)years and 33 (32–34) years, respectively (Table 1).

**Table 1:** Demographic characteristics of patients and health care professionals.

	Patients n (%); (n = 120)	HCPs n (%); (n = 30)
**A. Demographic characteristics**		
Gender		
Male	62 (51.7)	9 (30.0)
Female	58 (48.3)	21 (70.0)
Median age (years) [IQR]	63 [57–69]	33 [32–34]
Highest level of education obtained		
Primary	6 (5.0)	0
Secondary	61 (50.8)	0
Pre-university	32 (26.7)	0
Tertiary/Postgraduate	21 (17.5)	30 (100)
Median total years of working experience as a doctor [IQR]		8.0 [7.0–9.0]
Currently working	42 (35.0)	
Total household income per month		
<RM1000 (USD 243)	43 (35.8)	
RM1000 –RM3000(USD 243–730)	42 (35.0)	
RM3001 –RM5000(USD 730–1216)	16 (13.3)	
RM5001 –RM 10,000(USD 1216–2432)	12 (10.0)	
> RM10,000 (USD 2432)	7 (5.8)	
**B. Clinical data**		
Median duration patients diagnosed with DM (years) [IQR]	10.0 [5.0–16.0]	
Median HbA1C (%) [IQR]	7.5 [6.6–9.3]	
Presence of co-morbidities	98 (81.7)	
Median number of co-morbidities [IQR]	2.0 [1.0–2.0]	
Types of co-morbidities		
High blood pressure	75 (62.5)	
High cholesterol	74 (61.7)	
Heart problems	24 (20.0)	

IQR, Interquartile range; USD, United States Dollar, N, number, DM, Diabetes mellitus.

Thirty (25%) patients reported that they were unable to take their medications as instructed for the past two weeks (Table 2). The two main reasons reported were that they simply forgot (53.3%) or that they ran out of medications (33.3%).

**Table 2:** Medication adherence of patients and their reasons for non-adherence.

	n (%)
Number of patients who were unable to take their medications as directed for the past 2 weeks	30 (25.0)
Reason(s) for not taking medications as directed for the past 2 weeks*	
a) Simply forgot	16 (53.3)
b) Ran out of medicine(s)	10 (33.3)
c) Wanted to avoid side effects	5 (16.7)
d) Had too many medicines to take	5 (16.7)
e) Had stomach upset when I took the medicine(s) before food	5 (16.7)
f) Had to take medicines too frequently	3 (10.0)
g) Too expensive	3 (10.0)
h) Felt that the medicine(s) was (were) harmful	2 (6.7)
i) Felt healthy or better, so thought that I did not have to take my medicine(s)	2 (6.7)
j) I was confused about how to take my medicine(s)	2 (6.7)
k) When I travel	2 (6.7)
l) No private place for me to take my medicine(s)	1 (3.3)
m) Did not want other people to notice that I was taking medicines	1 (3.3)
n) Cannot see or feel whether my medicine(s) is(are) helping me	1 (3.3)
o) Too busy at work	1 (3.3)
p) Missed appointment	1 (3.3)
q) I drank alcohol	1 (3.3)

*patients were able to select more than one answer.

### Validity

#### Exploratory factor analysis

**Fig 1:**
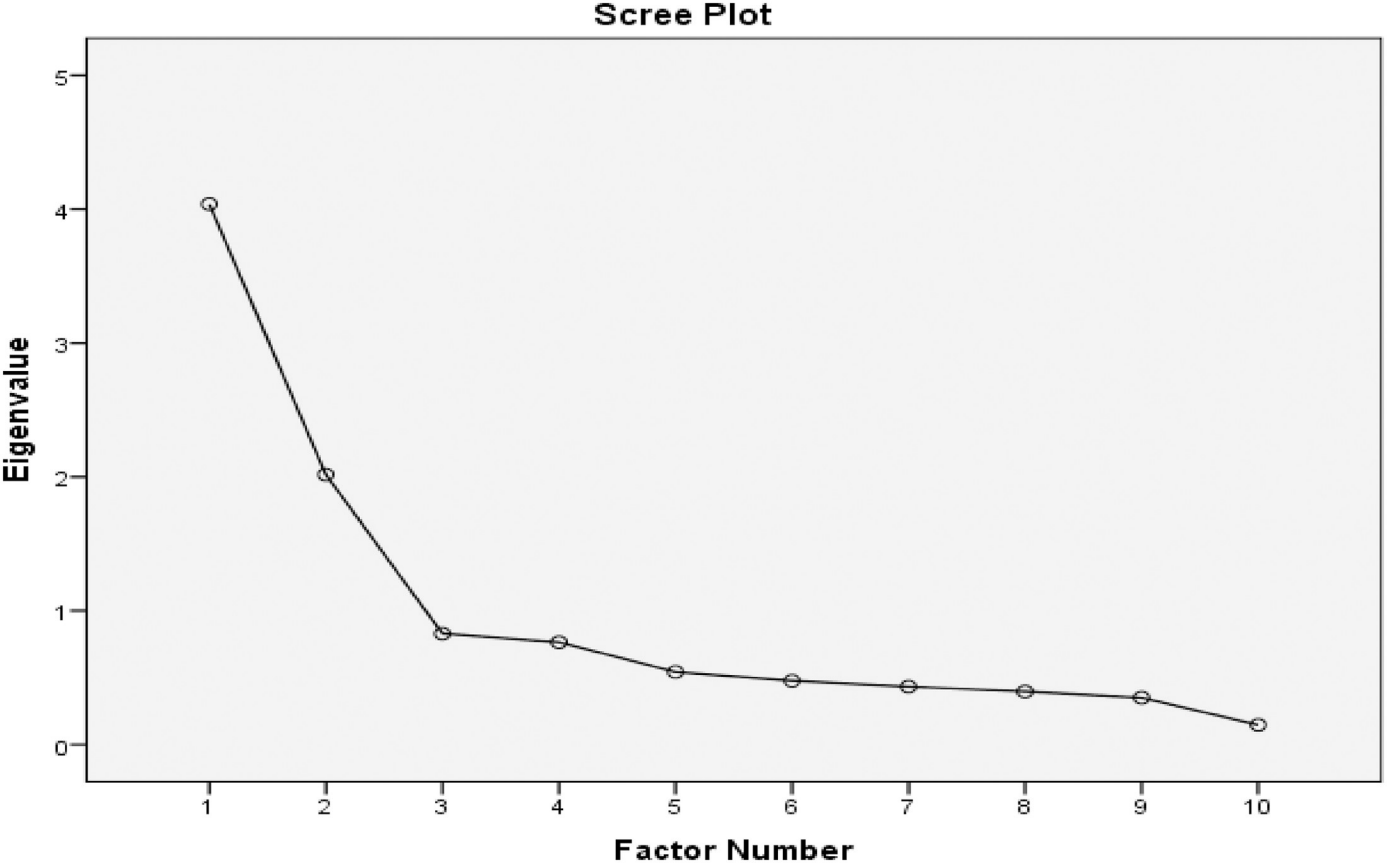
The number of constructs in the Patient-Medication Adherence Instrument (P-MAI).

*The Patient-Medication Adherence Instrument (P-MAI)*. EFA initially showed that the P-MAI-12 was a 3-factor model, with acceptable sampling adequacy (KMO = 0.798, Barlett’s test of sphericity: X^2^ = 542.0; df = 66; p-value < 0.001). Anti-image correlation matrix coefficients values were <0.5. All communality values were >0.3 except for item no. 11 and 12. Hence, both problematic items were removed. The number of factors was selected to be rotated as two (Fig 1), the loading factors were fixed as 0.4, and promax rotation was selected. We did this reiteratively and removed one problematic item (items no. 4) as its factor loading were < 0.4. Finally, the P-MAI-9 became a 2-factor model with two domains (“adherence” and “knowledge and belief”) and with 9 items [Table 3 and S2 Table]. This model explained 62.9% of the total variance.

**Table 3:** The exploratory factor analysis and concurrent validity of the Patient-Medication Adherence Instrument-9 (P-MAI-9) and Healthcare Professional-Medication Adherence Instrument-9 (H-MAI-9).

Exploratory factor analysis									Concurrent validity: Mann Whitney U test		
Domain	P-MAI-9				H-MAI-9				P-MAI-9; Median [IQR]	H-MAI-9; Median [IQR]	p-value
	No	Items	Factor loadings		No	Items	Factor loadings				
			1	2			1	2			
Knowledge and belief	9	I know why I am taking my medication(s) (eg. indication)	0.859		9	My patient knows why he/she is taking their medication(s) (eg. indication)	0.830		4.0 [4.0–4.0]	4.0 [4.0–5.0]	0.950
	8	I know how to take my medication(s) (eg. dose, frequency)	0.826		8	My patient knows how to take his/her medication(s) (eg. dose, frequency)	0.747		4.0 [4.0–4.0]	4.0 [4.0–4.0]	0.317
	7	I am able to make a decision together with my doctor regarding the medication(s) that have been given to me	0.767		7	My patient is able to make a decision together with his/her doctor regarding his/her medication(s)	0.911		4.0 [4.0–4.0]	4.0 [4.0–4.0]	0.644
	5	I am confident that my medication(s) are helping me	0.740		5	My patient is confident that his/her medication(s) are helping him/her	0.732		4.0 [4.0–4.0]	4.0 [4.0–4.0]	0.093
	6	I am satisfied with the information that my doctor has shared with me	0.676		6	My patient is satisfied with the information shared by his/her doctor	0.699		4.0 [4.0–4.0]	4.0 [4.0–4.0]	0.494
	4	I have a good understanding of my illness	0.520		4	My patient has a good understanding of his/her illness	0.837		4.0 [4.0–4.0]	4.0 [3.3–4.0]	0.009*
	1	I take my medication(s) everyday as directed	0.534		1	My patient is taking his/her medication(s) everyday as directed	0.588		4.0 [4.0–4.0]	4.0 [4.0–5.0]	0.579
Total score of knowledge and belief domain									28.0 [28.0–29.0]	28.0 [26.0–30.0]	0.082
Adherence	3	I do not take medication(s) LESS than directed		0.761	3	My patient does not take his/her medication(s) LESS than directed		0.778	4.0 [4.0–4.0]	4.0 [2.0–4.8]	0.952
	2	I do not take medication(s) MORE than directed		0.673	2	My patient does not take his/her medication(s) MORE than directed		0.839	4.0 [4.0–4.0]	4.0 [4.0–5.0]	0.479
Total score of adherence domain									8.0 [8.0–8.0]	8.0 [6.0–8.8]	0.615
Deleted items**	4	I do not skip or stop taking medication(s) without informing my doctor			4	My patient does not skip or stop taking his/her medication(s)					
	11	I know about the side effects of my medication(s)			11	My patient is aware about the side effects of his/her medication(s)					
	12	I know how to contact the doctor/pharmacist or nurse regarding my medication(s)			12	When in doubt, my patient knows how to contact the doctor/pharmacist or nurse regarding his/her medication(s)					

*Statistically significant = p-value<0.05;

**Items number listed in this table is according to the P-MAI-12 and H-MAI-12.

**Fig 2:**
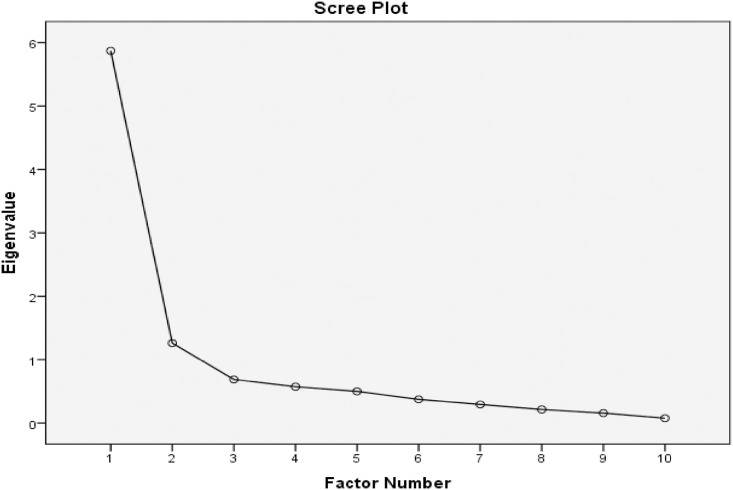
The number of constructs in the Healthcare Professional-Medication Adherence Instrument (H-MAI).

*The Healthcare Professional-Medication Adherence Instrument (H-MAI)*. Initially, EFA showed that the H-MAI-12 was a 3-factor model, with acceptable sampling adequacy (KMO = 0.870, Barlett’s test of sphericity: X^2^ = 1020.4; df = 66; p-value < 0.001). Anti-image correlation matrix coefficients values were <0.5. All communality values were >0.3 except for item no. 11 and 12. Both problematic items were then removed. The number of factors was selected to be rotated and fixed as two (Fig 2), the loading factors were fixed as 0.4, and promax rotation was selected. We did this reiteratively and removed one problematic item (items no. 4) as its factor loading were < 0.4. Finally, the H-MAI-9 became a 2-factor model with two domains (“adherence” and “knowledge and belief”) and with 9 items [Table 3 and S3 Table]. This model explained 71.2% of the total variance.

#### Concurrent validity

The total score of the P-MAI-9 and the H-MAI-9, as well as 8 out of 9 items were not significantly different (p = 0.091) [Table 3].

#### Flesch reading ease

Flesch reading ease for both the P-MAI-9 and the H-MAI-9 was 78.7 and 62.6, respectively.

### Reliability

#### Internal consistency

Reliability analysis was performed on the remaining 9 items (Table 4). The Cronbach’s alpha values for the P-MAI and H-MAI were 0.722 and 0.895, respectively. The Cronbach’s alpha value for the knowledge and belief domain was: P-MAI-9 = 0.860 and H-MAI-9 = 0.917; whilst for the adherence domain was: P-MAI-9 = 0.670 and HMAI-9 = 0.722. The corrected item-total correlations of all items in both instruments were >0.2.

**Table 4:** Reliability of the PATIENT-Medication Adherence Instrument (P-MAI-9) and HEALTHCARE PROFESSIONAL-Medication Adherence Instrument (H-MAI-9).

Item	Domains	Cronbach α	Corrected item-total correlation	Cronbach α if item deleted	Test (n = 120)		Retest (n = 87)		Wilcoxon signed-rank test p-value
					Median [IQR]	Mean [SD]	Median [IQR]	Mean [SD]	
A) PATIENT-Medication Adherence Instrument (P-MAI-9)									
1	Knowledge and belief	0.860	0.473	0.864	4.0 [4.0–4.0]	4.10 [0.58]	4.0 [4.0–4.0]	4.10 [0.42]	0.870
4			0.470	0.864	4.0 [4.0–4.0]	4.12 [0.57]	4.0 [4.0–4.0]	4.16 [0.40]	0.873
5			0.687	0.832	4.0 [4.0–4.0]	4.07 [0.56]	4.0 [4.0–4.0]	4.11 [0.23]	0.480
6			0.630	0.841	4.0 [4.0–4.0]	4.06 [0.50]	4.0 [4.0–4.0]	4.03 [0.23]	0.580
7			0.713	0.828	4.0 [4.0–4.0]	4.04 [0.57]	4.0 [4.0–4.0]	3.90 [0.37]	<0.001*
8			0.740	0.827	4.0 [4.0–4.0]	4.18 [0.47]	4.0 [4.0–4.0]	4.28 [0.46]	0.086
9			0.772	0.826	4.0 [4.0–4.0]	4.15 [0.42]	4.0 [4.0–4.0]	4.18 [0.46]	0.695
	Total score of knowledge and belief domain				28.0 [28.0–29.0]	28.7 [2.71]	28.0 [28.0–29.0]	28.8 [2.10]	0.879
2	Adherence	0.670	0.510	-	4.0 [4.0–4.0]	3.95 [0.78]	4.0 [4.0–4.0]	4.04 [0.29]	0.685
3			0.510	-	4.0 [4.0–4.0]	3.71 [0.93]	4.0 [4.0–4.0]	3.98 [0.45]	0.009*
	Total score of adherence domain				8.0 [8.0–8.0]	7.67 [1.49]	8.0 [8.0–8.0]	8.02 [0.64]	0.118
	Total score of both domains				36.0 [35.0–37.0]	36.4 [3.09]	36.0 [36.0–38.0]	36.8 [2.42]	0.383
B) HEALTHCARE PROFESSIONAL-Medication Adherence Instrument (H-MAI-9)									
1	Knowledge and belief	0.917	0.746	0.909	4.0 [4.0–5.0]	3.84 [1.16]	4.0 [4.0–5.0]	4.28 [0.79]	0.004*
4			0.809	0.898	4.0 [3.3–4.0]	3.79 [0.96]	4.0 [4.0–5.0]	4.18 [0.66]	0.001*
5			0.743	0.905	4.0 [4.0–4.0]	3.83 [0.94]	4.0 [4.0–5.0]	4.20 [0.64]	0.003*
6			0.552	0.922	4.0 [4.0–4.0]	4.01 [0.69]	4.0 [4.0–5.0]	4.20 [0.55]	0.079
7			0.698	0.910	4.0 [4.0–4.0]	3.99 [0.73]	4.0 [4.0–5.0]	4.18 [0.58]	0.049*
8			0.871	0.892	4.0 [4.0–4.0]	4.00 [0.87]	4.0 [4.0–5.0]	4.29 [0.66]	0.023*
9			0.855	0.893	4.0 [4.0–4.0]	4.01 [0.90]	4.0 [4.0–5.0]	4.28 [0.64]	0.037*
	Total score of knowledge and belief domain				28.0 [26.0–30.0]	27.5 [5.17]	28.0 [28.0–33.0]	29.6 [3.61]	<0.001
2	Adherence	0.722	0.569	-	4.0 [4.0–5.0]	3.88 [1.12]	4.0 [4.0–5.0]	4.28 [0.78]	0.004*
3			0.569	-	4.0 [2.0–4.8]	3.58 [1.27]	4.0 [4.0–5.0]	4.11 [0.99]	0.006*
	Total score of adherence domain				8.0 [6.0–8.8]	7.46 [2.13]	8.0 [8.0–10.0]	8.40 [1.67]	0.002
	Total score of both domains				36 [32.5–38.0]	34.93 [6.49]	36.0 [36.0–43.0]	38.00 [4.90]	<0.001*

*Statistically significant = p-value<0.05.

#### Test-retest

Test-retest reliability was assessed in 87/120 patients after a 2-week interval where 33 patients were lost to follow up (response rate = 72.5%) (Table 4). Seven out of nine items in the P-MAI-9 showed no significant difference between test and retest whereas 8/9 items in the H-MAI showed significant difference at test and retest (p>0.05).

### Sensitivity and specificity

The sensitivity and specificity of the P-MAI-9 were 37.8% and 51.6%, respectively whilst the sensitivity and specificity of the H-MAI-9 were 46.8% and 66.7%, respectively. (Table 5). The positive predictive values for the P-MAI-9 and HMAI-9 were 31/46 (67.4%) and 29/46 (63.0%), respectively. This means that the P-MAI-9 and HMAI-9 were able to correctly predict good glycaemic control in 67.4% and 63.0%, respectively of patients who were adherent to their medications. On the other hand, the negative predictive values of P-MAI-9 and H-MAI-9 were calculated as 16/67 (23.9%) and 34/67 (50.7%), respectively; which meant that P-MAI-9 and H-MAI-9 were able to correctly predict poor glycaemic control in 23.9% and 50.7%, respectively of patients who were non-adherent to their medications.

**Table 5:** Sensitivity and specificity of the PATIENT-Medication Adherence Instrument (P-MAI-9) and HEALTHCARE PROFESSIONAL-Medication Adherence Instrument (H-MAI-9).

	P-MAI-9 (n = 113)			H-MAI-9 (n = 113)		
Adherence	Adherence; n (%)	Non-adherence; n (%)	Positive and negative predictive value	Adherence; n (%)	Non-adherence; n (%)	Positive and negative predictive value
Good control of HbA1c (≤7%)	31 (37.8) [TP]	15 (48.4) [FP]	Positive PV = TP/(TP+FP) x 100% = 67.4%	29 (46.8) [TP]	17 (33.3) [FP]	Positive PV = TP/(TP+FP) x 100% = 63.0%
Poor control of HbA1c (>7%)	51 (62.2) [FN]	16 (51.6) [TN]	Negative PV = TN/(TN+FN) x 100% = 23.9%	33 (53.2) [FN]	34 (66.7) [TN]	Negative PV = TN/(TN+FN) x 100% = 50.7%
Sensitivity and specificity	Sensitivity TP/(TP+FN) x 100% = 37.8%	Specificity TN/(TN+FP) x 100% = 51.6%		Sensitivity TP/(TP+FN) x 100% = 46.8%	Specificity TN/(TN+FP) x 100% = 66.7%	

PV, Predictive value; TP, True positive; TN, True negative; FP, False positive; FN, False negative.

## Discussion

Our study found that the P-MAI-9 and the H-MAI-9 were able to assess medication adherence from the patient’s and their HCP’s perspectives, as they had adequate psychometric properties. The P-MAI-9 was found to be reliable, but the H-MAI-9 was not found to be reliable when the retest was conducted over the phone. The final version of both instruments consists of 9 items with two domains measuring “knowledge and belief” (7 items) and “adherence” (2 items).

The final P-MAI-9 and H-MAI-9 consist of 9 Likert-like items that measured patient’s and HCP’s perspectives on knowledge and understanding about their illnesses and medications (“knowledge and belief”), and their adherence to medication (“adherence”). These items were similar to previous studies which included items that either obtained information regarding the patient’s medication-taking behaviour and/or attempts to identify barriers to good medication-taking behaviour or beliefs associated with adherence [11]. Our instrument can be used to measure patient’s medication-taking behaviour and identify reasons for a patient’s non-adherence by identifying patient’s knowledge and belief towards their disease and medication from two different perspectives (i.e patient’s and HCP’s perspectives). However, the P-MAI-9 and H-MAI-9 were only able to assess patient’s medication adherence in the implementation process [5], as the majority of patients have been taking their oral hypoglycaemic agents for a median duration of 10 years.

The P-MAI-9 and the H-MAI-9 have Flesh reading ease scores of more than 60; indicating that both instruments were easily understood by those who have completed lower secondary education (>15 years old) or the equivalent of 8^th^ to 9^th^-grade studies in the United States [73]. The majority of the participants (patients = 95%; HCPs = 100%) had completed secondary school education (>11 years of education) indicating that both groups of participants were able to complete the self-administered instrument without any problems.

The total score of the P-MAI-9 and the H-MAI-9 were not found to be significantly different, indicating that medication adherence assessed by the patient and the HCP were similar. This shows that the patient’s medication adherence could be assessed using the P-MAI-9 and the results obtained could be confirmed using the H-MAI-9. These instruments may provide researchers with an alternative tool for assessing medication adherence more accurately.

The overall Cronbach’s alpha and the individual domains of the P-MAI-9 and the H-MAI-9 were >0.6, indicating acceptable internal consistency [74]. At test-retest, 7/9 items in the P-MAI-9 showed no significant difference indicating that the instrument has achieved stable reliability. In contrast, 8/9 items in the H-MAI showed a significant difference between test and retest. The medication adherence total score assessed by HCP at test (median = 36; mean = 35 and at retest was (median = 36; mean = 38) [Table 4]. Eight out of nine items in H-MAI-9 were significantly higher at retest (p<0.05). At retest, medication adherence scores were higher than at test. This may be because test was conducted during a doctor-patient consultation, where doctors were able to assess their patient’s medication adherence based on the patient’s body language, clinical and laboratory results; whilst retest was conducted. over-the-phone [78], and patients may tend to report “better” adherence in fear of “displeasing” their doctor [79]. This suggests that the H-MAI-9 should only be used in a clinical setting, and not over the phone.

The sensitivity and specificity of the P-MAI-9 and H-MAI-9 were found to be similar. This shows that both instruments almost mirrored each other. The specificity of the P-MAI-9 and the H-MAI-9 in identifying patients were non-adherent to their medications and had poor glycaemic control were 51.6% and 66.7%, respectively. However, it was found that the sensitivity of P-MAI-9 and H-MAI-9 which correctly determining participants who were adherent and also had good glycaemic control were only 37.8% and 46.8%, respectively. Similar to a previous study conducted in Thailand, the low sensitivity and high specificity of the P-MAI-9 and the H-MAI-9 suggesting that both instruments can be used to identify patients with poor adherence in clinical practice [80]. Besides, the low sensitivity and high specificity of the P-MAI-9 and the H-MAI-9 were probably attributed to overestimated adherence behaviour when compared to other assessments [60]. Studies have shown that patients only tend to be truthful about their non-adherence to medication when they know that they will not be criticized [81, 82]. This is possible when HCPs manage to develop a good rapport with their patients during patient care.

One of the limitations of this study was that the P-MAI and H-MAI were both developed in English. This means that these instruments can only be used by patients and HCPs who understand English. Convergent validity was not performed, as there were no validated instruments to assess medication adherence from two different perspectives (patient’s and HCP’s perspectives) at the time this study was conducted. We did not perform CFA as CFA is usually assessed for instruments that have a well-developed underlying theory for hypothesized patterns of loadings [83]. Ideally, the retest of P-MAI and H-MAI should be conducted in a clinical setting and not via telephone interviews. However, this could not be done as it was almost impossible to ask patients to come back after two weeks just to assess the reliability of the instruments.

## Conclusion

The P-MAI-9 and H-MAI-9 were developed to assess medication adherence among people with diabetes mellitus in Malaysia from the patient’s and HCP’s perspectives, respectively. The final version of the P-MAI-9 and H-MAI-9 which consists of 9 items with two domains measuring “knowledge and belief” (7 items) and “adherence” (2 items) had low sensitivity and high specificity, suggesting that both instruments can be used for identifying patients who are likely to be non-adherent to their medications in clinical practice.

## Supporting information

S1 Table Psychometric properties of validated self-reported adherence instruments. (DOCX)

S2 Table The PATIENT-Medication Adherence Instrument (PMAI-9). (DOCX)

S3 Table The HEALTHCARE-PROFESSIONAL-Medication Adherence Instrument (H-MAI-9). (DOCX)

## References

[1] Gellad WF, Grenard JL, Marcum ZA. A Systematic Review of Barriers to Medication Adherence in the Elderly: Looking Beyond Cost and Regimen Complexity. The American journal of geriatric pharmacotherapy. 2011;9(1):11–23. 10.1016/j.amjopharm.2011.02.004 21459305 PMC3084587

[2] Tsai KT, Chen JH, Wen CJ, Kuo HK, Lu IS, Chiu LS, et al Medication adherence among geriatric outpatients prescribed multiple medications. Am J Geriatr Pharmacother. 2012;10(1):61–8. 10.1016/j.amjopharm.2011.11.005 22264853

[3] Shruthi R, Jyothi R, Pundarikaksha HP, Nagesh GN, Tushar TJ. A Study of Medication Compliance in Geriatric Patients with Chronic Illnesses at a Tertiary Care Hospital. Journal of Clinical and Diagnostic Research: JCDR. 2016;10(12):FC40–FC3. 10.7860/JCDR/2016/21908.9088 28208878 PMC5296451

[4] Osterberg L, Blaschke T. Adherence to medication. The New England journal of medicine. 2005;353(5):487–97. 10.1056/NEJMra050100 16079372

[5] Vrijens B, De Geest S, Hughes DA, Przemyslaw K, Demonceau J, Ruppar T, et al A new taxonomy for describing and defining adherence to medications. 2012;73(5):691–705. 10.1111/j.1365-2125.2012.04167.x PMC3403197 22486599

[6] van Dijk L, Heerdink ER, Somai D, van Dulmen S, Sluijs EM, de Ridder DT, et al Patient risk profiles and practice variation in nonadherence to antidepressants, antihypertensives and oral hypoglycemics. BMC Health Services Research. 2007;7(1):51 10.1186/1472-6963-7-51 17425792 PMC1855317

[7] Hayes TL, Hunt JM, Adami A, Kaye JA, editors. An electronic pillbox for continuous monitoring of medication adherence. Engineering in Medicine and Biology Society, 2006 EMBS'06 28th Annual International Conference of the IEEE; 2006: IEEE. 10.1109/IEMBS.2006.260367 PMC2911441 17946369

[8] Kronish IM, Ye S. Adherence to cardiovascular medications: lessons learned and future directions. Progress in cardiovascular diseases. 2013;55(6):590–600. 10.1016/j.pcad.2013.02.001 23621969 PMC3639439

[9] Lam WY, Fresco P. Medication adherence measures: an overview. BioMed Research International. 2015;2015 10.1155/2015/217047 26539470 PMC4619779

[10] Kleppe M, Lacroix J, Ham J, Midden C. The development of the ProMAS: a Probabilistic Medication Adherence Scale. Patient preference and adherence. 2015;9:355–67. 10.2147/PPA.S76749 25784791 PMC4356448

[11] Nguyen TMU, Caze AL, Cottrell N. What are validated self‐report adherence scales really measuring?: a systematic review. British journal of clinical pharmacology. 2014;77(3):427–45. 23803249 10.1111/bcp.12194 PMC3952718

[12] Kalichman SC, Amaral CM, Swetzes C, Jones M, Macy R, Kalichman MO, et al A simple single-item rating scale to measure medication adherence: further evidence for convergent validity. Journal of the International Association of Physicians in AIDS Care (Chicago, Ill: 2002). 2009;8(6):367–74. 10.1177/1545109709352884 19952289 PMC3015098

[13] Byerly MJ, Nakonezny PA, Rush AJ. The Brief Adherence Rating Scale (BARS) validated against electronic monitoring in assessing the antipsychotic medication adherence of outpatients with schizophrenia and schizoaffective disorder. Schizophr Res. 2008;100(1–3):60–9. 10.1016/j.schres.2007.12.470 18255269

[14] Barroso PF, Schechter M, Gupta P, Bressan C, Bomfim A, Harrison LH. Adherence to antiretroviral therapy and persistence of HIV RNA in semen. Journal of acquired immune deficiency syndromes (1999). 2003;32(4):435–40. 10.1097/00126334-200304010-00014 12640203

[15] Mannheimer SB, Mukherjee R, Hirschhorn LR, Dougherty J, Celano SA, Ciccarone D, et al The CASE adherence index: A novel method for measuring adherence to antiretroviral therapy. AIDS care. 2006;18(7):853–61. 10.1080/09540120500465160 16971298 PMC2567829

[16] Gehi AK, Ali S, Na B, Whooley MA. Self-reported medication adherence and cardiovascular events in patients with stable coronary heart disease: The heart and soul study. Archives of Internal Medicine. 2007;167(16):1798–803. 10.1001/archinte.167.16.1798 17846400 PMC2789555

[17] Grymonpre RE, Didur CD, Montgomery PR, Sitar DS. Pill count, self-report, and pharmacy claims data to measure medication adherence in the elderly. Annals of Pharmacotherapy. 1998;32(7–8):749–54. 10.1345/aph.17423 9681089

[18] Kerr T, Hogg RS, Yip B, Tyndall MW, Montaner J, Wood E. Validity of self-reported adherence among injection drug users. Journal of the International Association of Physicians in AIDS Care. 2008;7(4):157–9. 10.1177/1545109708320686 18626123

[19] Willey C, Redding C, Stafford J, Garfield F, Geletko S, Flanigan T, et al Stages of change for adherence with medication regimens for chronic disease: development and validation of a measure. Clinical therapeutics. 2000;22(7):858–71. 10.1016/s0149-2918(00)80058-2 10945512

[20] Kripalani S, Risser J, Gatti ME, Jacobson TA. Development and evaluation of the Adherence to Refills and Medications Scale (ARMS) among low-literacy patients with chronic disease. Value in health: the journal of the International Society for Pharmacoeconomics and Outcomes Research. 2009;12(1):118–23. 10.1111/j.1524-4733.2008.00400.x 19911444

[21] Hahn SR, Park J, Skinner EP, Yu-Isenberg KS, Weaver MB, Crawford B, et al Development of the ASK-20 adherence barrier survey. Current medical research and opinion. 2008;24(7):2127–38. 10.1185/03007990802174769 18554431

[22] Svarstad BL, Chewning BA, Sleath BL, Claesson C. The Brief Medication Questionnaire: a tool for screening patient adherence and barriers to adherence. Patient education and counseling. 1999;37(2):113–24. 10.1016/s0738-3991(98)00107-4 14528539

[23] Brooks CM, Richards JM, Kohler CL, Soong SJ, Martin B, Windsor RA, et al Assessing adherence to asthma medication and inhaler regimens: a psychometric analysis of adult self-report scales. Medical care. 1994;32(3):298–307. 10.1097/00005650-199403000-00008 8145604

[24] Choo PW, Rand CS, Inui TS, Lee ML, Cain E, Cordeiro-Breault M, et al Validation of patient reports, automated pharmacy records, and pill counts with electronic monitoring of adherence to antihypertensive therapy. Medical care. 1999;37(9):846–57. 10.1097/00005650-199909000-00002 10493464

[25] Godin G, Gagne C, Naccache H. Validation of a self-reported questionnaire assessing adherence to antiretroviral medication. AIDS patient care and STDs. 2003;17(7):325–32. 10.1089/108729103322231268 12952734

[26] Kim MT, Hill MN, Bone LR, Levine DM. Development and testing of the Hill-Bone Compliance to High Blood Pressure Therapy Scale. Progress in cardiovascular nursing. 2000;15(3):90–6. 10951950 10.1111/j.1751-7117.2000.tb00211.x

[27] Chisholm MA, Lance CE, Williamson GM, Mulloy LL. Development and validation of the immunosuppressant therapy adherence instrument (ITAS). Patient education and counseling. 2005;59(1):13–20. 10.1016/j.pec.2004.09.003 16198214

[28] Clayton CD, Veach J, Macfadden W, Haskins J, Docherty JP, Lindenmayer JP. Assessment of clinician awareness of nonadherence using a new structured rating scale. Journal of psychiatric practice. 2010;16(3):164–9. 10.1097/01.pra.0000375712.85454.c6 20485104

[29] Morisky DE, Ang A, Krousel-Wood M, Ward HJ. Predictive validity of a medication adherence measure in an outpatient setting. Journal of clinical hypertension (Greenwich, Conn). 2008;10(5):348–54. 10.1111/j.1751-7176.2008.07572.x PMC2562622 18453793

[30] Reynolds K, Viswanathan HN, O’Malley CD, Muntner P, Harrison TN, Cheetham TC, et al Psychometric properties of the Osteoporosis-specific Morisky Medication Adherence Scale in postmenopausal women with osteoporosis newly treated with bisphosphonates. The Annals of pharmacotherapy. 2012;46(5):659–70. 10.1345/aph.1Q652 22510666

[31] Martinez CER, Sossa MP, Rand CS. Validation of a questionnaire for assessing adherence to metered-dose inhaler use in asthmatic children. Pediatric Asthma, Allergy & Immunology. 2007;20(4):243–54.

[32] Lewis SJ, Abell N. Development and Evaluation of the Adherence Attitude Inventory. Research on Social Work Practice. 2002;12(1):107–23.

[33] Morisky DE, Green LW, Levine DM. Concurrent and predictive validity of a self-reported measure of medication adherence. Medical care. 1986;24(1):67–74. 10.1097/00005650-198601000-00007 3945130

[34] Ogedegbe G, Mancuso CA, Allegrante JP, Charlson ME. Development and evaluation of a medication adherence self-efficacy scale in hypertensive African-American patients. Journal of clinical epidemiology. 2003;56(6):520–9. 10.1016/s0895-4356(03)00053-2 12873646

[35] Fernandez S, Chaplin W, Schoenthaler AM, Ogedegbe G. Revision and validation of the medication adherence self-efficacy scale (MASES) in hypertensive African Americans. Journal of behavioral medicine. 2008;31(6):453–62. 10.1007/s10865-008-9170-7 18784996 PMC3763496

[36] Unni EJ, Farris KB. Development of a new scale to measure self-reported medication nonadherence. Research in social & administrative pharmacy: RSAP. 2015;11(3):e133–43. 10.1016/j.sapharm.2009.06.005 21272524

[37] Risser J, Jacobson TA, Kripalani S. Development and psychometric evaluation of the Self-efficacy for Appropriate Medication Use Scale (SEAMS) in low-literacy patients with chronic disease. Journal of nursing measurement. 2007;15(3):203–19. 10.1891/106137407783095757 18232619

[38] Hogan TP, Awad AG, Eastwood R. A self-report scale predictive of drug compliance in schizophrenics: reliability and discriminative validity. Psychological medicine. 1983;13(1):177–83. 10.1017/s0033291700050182 6133297

[39] Dolder CR, Lacro JP, Warren KA, Golshan S, Perkins DO, Jeste DV. Brief evaluation of medication influences and beliefs: development and testing of a brief scale for medication adherence. Journal of clinical psychopharmacology. 2004;24(4):404–9. 10.1097/01.jcp.0000130554.63254.3a 15232332

[40] Lee S, Bae YH, Worley M, Law A. Validating the modified drug adherence Work-Up (M-DRAW) Tool to identify and address barriers to medication adherence. Pharmacy. 2017;5(3):52 10.3390/pharmacy5030052 28970464 PMC5622364

[41] Schroeder K, Fahey T, Hay AD, Montgomery A, Peters TJ. Adherence to antihypertensive medication assessed by self-report was associated with electronic monitoring compliance. Journal of clinical epidemiology. 2006;59(6):650–1. 10.1016/j.jclinepi.2005.10.013 16713529

[42] Bell DJ, Kapitao Y, Sikwese R, van Oosterhout JJ, Lalloo DG. Adherence to antiretroviral therapy in patients receiving free treatment from a government hospital in Blantyre, Malawi. Journal of acquired immune deficiency syndromes (1999). 2007;45(5):560–3. 10.1097/QAI.0b013e3180decadb 17558333

[43] Jonsdottir H, Opjordsmoen S, Birkenaes AB, Engh JA, Ringen PA, Vaskinn A, et al Medication adherence in outpatients with severe mental disorders: relation between self-reports and serum level. Journal of clinical psychopharmacology. 2010;30(2):169–75. 10.1097/JCP.0b013e3181d2191e 20520290

[44] Fodor GJ, Kotrec M, Bacskai K, Dorner T, Lietava J, Sonkodi S, et al Is interview a reliable method to verify the compliance with antihypertensive therapy? An international central-European study. Journal of hypertension. 2005;23(6):1261–6. 10.1097/01.hjh.0000170390.07321.ca 15894903

[45] Horne R, Weinman J, Hankins M. The beliefs about medicines questionnaire: The development and evaluation of a new method for assessing the cognitive representation of medication. Psychology & Health. 1999;14(1):1–24.

[46] Munoz-Moreno JA, Fumaz CR, Ferrer MJ, Tuldra A, Rovira T, Viladrich C, et al Assessing self-reported adherence to HIV therapy by questionnaire: the SERAD (Self-Reported Adherence) Study. AIDS research and human retroviruses. 2007;23(10):1166–75. 10.1089/aid.2006.0120 17961100

[47] Knobel H, Alonso J, Casado JL, Collazos J, Gonzalez J, Ruiz I, et al Validation of a simplified medication adherence questionnaire in a large cohort of HIV-infected patients: the GEEMA Study. AIDS (London, England). 2002;16(4):605–13. 10.1097/00002030-200203080-00012 11873004

[48] Greaves CJ, Hyland ME, Halpin DM, Blake S, Seamark D. Patterns of corticosteroid medication use: non-adherence can be effective in milder asthma. Primary care respiratory journal: journal of the General Practice Airways Group. 2005;14(2):99–105. 10.1016/j.pcrj.2004.09.005 16701705 PMC6743549

[49] de Klerk E, van der Heijde D, Landewe R, van der Tempel H, van der Linden S. The compliance-questionnaire-rheumatology compared with electronic medication event monitoring: a validation study. The Journal of rheumatology. 2003;30(11):2469–75. 14677194

[50] Wetzels G, Nelemans P, van Wijk B, Broers N, Schouten J, Prins M. Determinants of poor adherence in hypertensive patients: development and validation of the "Maastricht Utrecht Adherence in Hypertension (MUAH)-questionnaire". Patient education and counseling. 2006;64(1–3):151–8. 10.1016/j.pec.2005.12.010 16427764

[51] Dima AL, van Ganse E, Laforest L, Texier N, de Bruin M, group tA-L. Measuring medication adherence in asthma: Development of a novel self-report tool. Psychology & health. 2017;32(10):1288–307. 10.1080/08870446.2017.1290248 28276742

[52] Chung WW, Chua SS, Lai PSM, Morisky DE. The Malaysian Medication Adherence Scale (MALMAS): Concurrent Validity Using a Clinical Measure among People with Type 2 Diabetes in Malaysia. PLoS ONE. 2015;10(4):e0124275 10.1371/journal.pone.0124275 25909363 PMC4409377

[53] Shima R, Farizah H, Majid HA. The 11-item Medication Adherence Reasons Scale: reliability and factorial validity among patients with hypertension in Malaysian primary healthcare settings. Singapore medical journal. 2015;56(8):460 10.11622/smedj.2015069 25902719 PMC4545136

[54] Najimi A, Mostafavi F, Sharifirad G, Golshiri P. Development and study of self-efficacy scale in medication adherence among Iranian patients with hypertension. Journal of education and health promotion. 2017;6 10.4103/jehp.jehp_64_16 29114551 PMC5651667

[55] George J, Mackinnon A, Kong DC, Stewart K. Development and validation of the Beliefs and Behaviour Questionnaire (BBQ). Patient education and counseling. 2006;64(1–3):50–60. 10.1016/j.pec.2005.11.010 16843634

[56] Thompson K, Kulkarni J, Sergejew AA. Reliability and validity of a new Medication Adherence Rating Scale (MARS) for the psychoses. Schizophr Res. 2000;42(3):241–7. 10.1016/s0920-9964(99)00130-9 10785582

[57] Lambert EV, Steyn K, Stender S, Everage N, Fourie JM, Hill M. Cross-cultural validation of the hill-bone compliance to high blood pressure therapy scale in a South African, primary healthcare setting. Ethnicity & disease. 2006;16(1):286–91. 16599385

[58] Matza LS, Park J, Coyne KS, Skinner EP, Malley KG, Wolever RQ. Derivation and validation of the ASK-12 adherence barrier survey. The Annals of pharmacotherapy. 2009;43(10):1621–30. 10.1345/aph.1M174 19776298

[59] Anghel LA, Farcas AM, Oprean RN. An overview of the common methods used to measure treatment adherence. Med Pharm Rep. 2019;92(2):117–22. 10.15386/mpr-1201 31086837 PMC6510353

[60] Stirratt MJ, Dunbar-Jacob J, Crane HM, Simoni JM, Czajkowski S, Hilliard ME, et al Self-report measures of medication adherence behavior: recommendations on optimal use. Translational behavioral medicine. 2015;5(4):470–82. 26622919 10.1007/s13142-015-0315-2 PMC4656225

[61] Jimmy B, Jose J. Patient medication adherence: measures in daily practice. Oman medical journal. 2011;26(3):155–9. 10.5001/omj.2011.38 22043406 PMC3191684

[62] Heeb RM, Kreuzberg V, Grossmann V. Physicians’ Assessment of Medication Adherence: A Systematic Review. J Pharma Care Health Sys. 2019;6(202):2376–0419.1000202.

[63] Meddings J, Kerr EA, Heisler M, Hofer TP. Physician assessments of medication adherence and decisions to intensify medications for patients with uncontrolled blood pressure: still no better than a coin toss. BMC Health Services Research. 2012;12(1):270 10.1186/1472-6963-12-270 22909303 PMC3570326

[64] Mirzaei A, Carter SR, Chen JY, Rittsteuer C, Schneider CR. Development of a questionnaire to measure consumers’ perceptions of service quality in community pharmacies. Research in Social and Administrative Pharmacy. 2019;15(4):346–57. 10.1016/j.sapharm.2018.05.005 29903653

[65] Grew B, Schneider CR, Mirzaei A, Carter SR. Validation of a questionnaire for consumers’ perception of service quality in community pharmacy. Research in Social and Administrative Pharmacy. 2019;15(6):673–81. 10.1016/j.sapharm.2018.08.008 30170902

[66] Abdullah A, Liew SM, Hanafi NS, Ng CJ, Lai PSM, Chia YC, et al What influences patients’ acceptance of a blood pressure telemonitoring service in primary care? A qualitative study. Patient preference and adherence. 2016;10:99–106. 10.2147/PPA.S94687 26869773 PMC4734809

[67] McMillan SS, King M, Tully MP. How to use the nominal group and Delphi techniques. Int J Clin Pharm. 2016;38(3):655–62. 10.1007/s11096-016-0257-x 26846316 PMC4909789

[68] Thirusanku J, Yunus MM. The many faces of Malaysian English. ISRN Education. 2012;2012.

[69] Schechter CB, Walker EA. Improving adherence to diabetes self-management recommendations. Diabetes Spectrum. 2002;15(3):170–5.

[70] Sherwani SI, Khan HA, Ekhzaimy A, Masood A, Sakharkar MK. Significance of HbA1c Test in Diagnosis and Prognosis of Diabetic Patients. Biomark Insights. 2016;11:95–104. 10.4137/BMI.S38440 27398023 PMC4933534

[71] Osborne JW, Costello AB. Sample size and subject to item ratio in principal components analysis. 2004;9(1):11.

[72] Costello AB, Osborne J. Best practices in exploratory factor analysis: Four recommendations for getting the most from your analysis. Practical assessment, research, and evaluation. 2005;10(1):7.

[73] Anoymous. The Flesch reading ease readibility formula. 2019.

[74] Ursachi G, Horodnic IA, Zait A. How reliable are measurement scales? External factors with indirect influence on reliability estimators. Procedia Economics and Finance. 2015;20:679–86.

[75] Pallant J. SPSS survival manual: A step by step guide to data analysis using IBM SPSS. 6th Edition ed: Routledge; 2016.

[76] Ministry of Health. Clinical Practive Guidelines Management of Type 2 Diabetes Mellitus (5th Edition). 2015.

[77] Lalkhen AG, McCluskey A. Clinical tests: sensitivity and specificity. Continuing Education in Anaesthesia Critical Care & Pain. 2008;8(6):221–3.

[78] van Galen LS, Car J. Telephone consultations. BMJ. 2018;360:k1047 10.1136/bmj.k1047 29599197

[79] Bezreh T, Laws MB, Taubin T, Rifkin DE, Wilson I. Challenges to physician–patient communication about medication use: a window into the skeptical patient’s world. Patient preference and adherence. 2012;6:11 10.2147/PPA.S25971 22272065 PMC3262486

[80] Okoli C, Pawlowski SD. The Delphi method as a research tool: an example, design considerations and applications. Information & Management. 2004;42(1):15–29.

[81] Martin LR, Williams SL, Haskard KB, Dimatteo MR. The challenge of patient adherence. Therapeutics and clinical risk management. 2005;1(3):189–99. 18360559 PMC1661624

[82] Sawada N, Uchida H, Watanabe K, Kikuchi T, Suzuki T, Kashima H, et al How successful are physicians in eliciting the truth from their patients? A large-scale Internet survey from patients’ perspectives. The Journal of clinical psychiatry. 2012;73(3):311–7. 10.4088/JCP.11m07078 22490259

[83] Hurley AE, Scandura TA, Schriesheim CA, Brannick MT, Seers A, Vandenberg RJ, et al Exploratory and confirmatory factor analysis: Guidelines, issues, and alternatives. Journal of organizational behavior. 1997:667–83.

