# Supplementary material for: Development of a PATIENT-Medication Adherence Instrument (P-MAI) and a HEALTHCARE PROFESSIONAL-Medication Adherence Instrument (H-MAI) using the nominal group technique
Source: PLoS One. 2020 Nov 11;15(11):e0242051. doi: 10.1371/journal.pone.0242051 (PMC7657514; doi:10.1371/journal.pone.0242051)
Supplement: S1 Table — (DOCX) [file pone.0242051.s001.docx]

**S1 Table: Psychometric properties of validated self-reported adherence instruments**

| No | Self-report adherence instruments | Country | No. of items | Psychometric properties | | | | | | |
| --- | --- | --- | --- | --- | --- | --- | --- | --- | --- | --- |
|  |  |  |  | Validity | | | | | Reliability | |
|  |  |  |  | Face validity | Content validity | Factor analysis | Correlation (criterion validity) | Other types | Internal consistency | Test-retest |
|  | Adherence Visual Analogue Scale (VAS) Computerized (1) | United States of America | 1 |  |  |  | X |  |  |  |
|  | Brief Adherence Rating Scale (BARS) (2) | United States of America | 4 |  |  |  | X | Concurrent validity | X | X |
|  | Barroso et al. 30-day Adherence Question (3) | United States of America | 1 |  |  |  | X |  |  |  |
|  | Centre for Adherence Support Evaluation (CASE) Adherence Index (4) | United States of America | 3 |  |  |  | X |  |  |  |
|  | Gehi et al. Adherence Question (5) | United States of America | 1 |  |  |  | X |  |  |  |
|  | Adherence to Refills and Medications Scale (ARMS) (6) | United States of America | 12 |  |  | X  (EFA) | X |  | X | X |
|  | Adherence Starts with Knowledge-12 (ASK-12) (7) | United States of America | 12 |  |  | X  (EFA) | X | Discriminant validity Convergent validity | X | X |
|  | Adherence Starts with Knowledge-20 (ASK-20) (8) | United States of America | 20 |  |  | X  (EFA) | X | Convergent validity  Concurrent validity | X |  |
|  | Brief Medication Questionnaire (9) | United States of America | 9 |  |  |  | X |  |  |  |
|  | Brooks Medication Adherence Scale (BMAS) (10) | United States of America | 4 |  |  |  | X |  | X |  |

**S1 Table: Psychometric properties of validated self-reported adherence instruments (continued)**

| No | Self-report adherence instruments | Country | No. of items | Psychometric properties | | | | | | |
| --- | --- | --- | --- | --- | --- | --- | --- | --- | --- | --- |
|  |  |  |  | Validity | | | | | Reliability | |
|  |  |  |  | Face validity | Content validity | Factor analysis | Correlation (criterion validity) | Other types | Internal consistency | Test-retest |
|  | Choo et al. 5-item Questionnaire (11) | United States of America | 5 |  |  |  | X |  |  |  |
|  | Hill-Bone Compliance Scale – 14 (12) | United States of America | 14 |  | X | X  (EFA) | X |  | X |  |
|  | Immunosuppressant Therapy Adherence Scale (ITAS) (13) | United States of America | 4 |  |  |  | X | Convergent validity | X |  |
|  | Medication Adherence Assessment Tool (MAAT) (14) | United States of America | 12 |  | X |  | X | Convergent validity | X |  |
|  | Morisky Medication Adherence Scale (MMAS) (15) | United States of America | 8 |  |  | X  (CFA) | X | Concurrent validity | X |  |
|  | Osteoporosis-Specific Morisky Medication Adherence Scale (OS-MMAS) (16) | United States of America | 8 |  |  | X  (CFA) | X | Convergent validity | X | X |
|  | Adherence Attitude Inventory (AAI) (17) | United States of America | 28 |  | X |  | X | Discriminant validity Convergent validity | X |  |
|  | Medication Adherence Questionnaire (MAQ) (18) | United States of America | 4 |  |  |  | X | Concurrent validity | X |  |
|  | Medication Adherence Self-Efficacy Scale (MASES) (19) | United States of America | 26 |  |  | X  (EFA) | X |  | X | X |
|  | Medication Adherence Self-Efficacy Scale Revised  (MASES-R) (20) | United States of America | 13 |  |  | X  (EFA, CFA) | X | Concurrent validity | X |  |

**S1 Table: Psychometric properties of validated self-reported adherence instruments (continued)**

| No | Self-report adherence instruments | Country | No. of items | Psychometric properties | | | | | | |
| --- | --- | --- | --- | --- | --- | --- | --- | --- | --- | --- |
|  |  |  |  | Validity | | | | | Reliability | |
|  |  |  |  | Face validity | Content validity | Factor analysis | Correlation (criterion validity) | Other types | Internal consistency | Test-retest |
|  | Medication Adherence Reasons Scale (21) | United States of America | 15 |  | X | X  (EFA) | X |  | X |  |
|  | The Self-Efficacy for Appropriate Medication Use Scale (SEAMS) (22) | United States of America | 13 |  |  | X  (EFA) | X |  | X | X |
|  | Brief Evaluation of Medication Influences and Beliefs (BEMIB) (23) | United States of America | 8 |  |  | X  (EFA) | X |  | X | X |
|  | Modified Drug Adherence Work-Up (M-DRAW) (24) | United States of America | 13 |  |  |  |  |  | X |  |
|  | Grymonpre et al. Adherence Question (25) | Canada | 1 |  |  |  | X |  |  |  |
|  | Kerr et al. Adherence Question (26) | Canada | 1 |  |  |  | X |  |  |  |
|  | Stages of Change for Adherence (SOCA) (27) | Canada | 2 |  |  |  | X | Construct validity |  |  |
|  | Godin et al. Self-Reported Adherence Questionnaire (28) | Canada | 6 |  |  |  | X |  |  |  |
|  | Drug Attitude Inventory (DAI) (29) | Canada | 30 |  |  | X  (EFA) | X | Discriminant validity |  | X |
|  | Pediatric Inhaler Adherence Questionnaire (PIAQ) (30) | Columbia | 6 | X | X |  | X | Convergent validity |  | X |
|  | Adherence Self-Report Questionnaire (ASRQ) (31) | United Kingdom | 1 |  |  |  | X |  |  |  |

**S1 Table: Psychometric properties of validated self-reported adherence instruments (continued)**

| No | Self-report adherence instruments | Country | No. of items | Psychometric properties | | | | | | |
| --- | --- | --- | --- | --- | --- | --- | --- | --- | --- | --- |
|  |  |  |  | Validity | | | | | Reliability | |
|  |  |  |  | Face validity | Content validity | Factor analysis | Correlation (criterion validity) | Other types | Internal consistency | Test-retest |
|  | Bell et al. Adherence Question (32) | United Kingdom | 1 |  |  |  | X |  |  |  |
|  | Reported Adherence to Medicine (RAM) Scale (33) | United Kingdom | 4 |  |  |  | X |  |  |  |
|  | The Patterns of Asthma Medication Use Questionnaire (34) | United Kingdom | 5 |  |  |  | X |  |  |  |
|  | Beliefs about Medicines Questionnaire (33) | United Kingdom | 18 |  |  | X  (EFA, CFA) | X | Discriminant validity | X |  |
|  | Medication Intake Survey-Asthma (MIS-A) (35) | France and United Kingdom | 9 |  |  |  |  | Discriminant validity Convergent validity |  |  |
|  | Compliance Questionnaire Rheumatology (CQR) (36) | Netherlands | 19 |  |  |  | X | Discriminant validity |  |  |
|  | Maastricht Utrecht Adherence in Hypertension (MUAH) Questionnaire (37) | Netherlands | 25 |  |  | X  (EFA) | X | Convergent validity | X | X |
|  | Probabilistic Medication Adherence Scale (ProMAS) (38) | Netherlands | 37 |  |  |  | X |  | X |  |
|  | Self-Reported Adherence (SERAD) Questionnaire (39) | Spain | 13 |  |  |  | X |  |  |  |
|  | Simplified Medication Adherence Questionnaire (SMAQ) (40) | Spain | 6 |  |  |  | X |  | X |  |

**S1 Table: Psychometric properties of validated self-reported adherence instruments (continued)**

| No | Self-report adherence instruments | Country | No. of items | Psychometric properties | | | | | | |
| --- | --- | --- | --- | --- | --- | --- | --- | --- | --- | --- |
|  |  |  |  | Validity | | | | | Reliability | |
|  |  |  |  | Face validity | Content validity | Factor analysis | Correlation (criterion validity) | Other types | Internal consistency | Test-retest |
|  | Medication Adherence Report Scale – 5 (MARS-5) (41) | Norway | 5 |  |  |  | X |  | X |  |
|  | Fodor et al. Adherence Questionnaire (42) | Austria, Hungary, and Slovakia | 9 |  |  |  | X |  |  |  |
|  | Medication Adherence Reasons Scale (MAR-Scale) (43) | Malaysia | 11 |  | X | X  (EFA, CFA) |  |  | X |  |
|  | Malaysian Medication Adherence Scale (MALMAS) (44) | Malaysia | 8 | X |  |  | X | Convergent Validity, Concurrent validity | X | X |
|  | Self‑efficacy scale in patients with hypertension (45) | Iran | 18 | X | X | X  (EFA) | X |  | X | X |
|  | Beliefs and Behaviour Questionnaire (BBQ) (46) | Australia | 30 | X | X | X  (EFA) | X | Discriminant validity Convergent validity | X | X |
|  | Medication Adherence Report Scale (MARS) (47) | Australia | 10 |  |  | X  (EFA) | X |  | X | X |
|  | Hill-Bone Compliance Scale – 10 (48) | South Africa | 10 |  |  |  | X |  | X |  |

**References**

1. Kalichman SC, Amaral CM, Swetzes C, Jones M, Macy R, Kalichman MO, et al. A simple single-item rating scale to measure medication adherence: further evidence for convergent validity. Journal of the International Association of Physicians in AIDS Care (Chicago, Ill : 2002). 2009;8(6):367-74.

2. Byerly MJ, Nakonezny PA, Rush AJ. The Brief Adherence Rating Scale (BARS) validated against electronic monitoring in assessing the antipsychotic medication adherence of outpatients with schizophrenia and schizoaffective disorder. Schizophr Res. 2008;100(1-3):60-9.

3. Barroso PF, Schechter M, Gupta P, Bressan C, Bomfim A, Harrison LH. Adherence to antiretroviral therapy and persistence of HIV RNA in semen. Journal of acquired immune deficiency syndromes (1999). 2003;32(4):435-40.

4. Mannheimer SB, Mukherjee R, Hirschhorn LR, Dougherty J, Celano SA, Ciccarone D, et al. The CASE adherence index: A novel method for measuring adherence to antiretroviral therapy. AIDS care. 2006;18(7):853-61.

5. Gehi AK, Ali S, Na B, Whooley MA. Self-reported medication adherence and cardiovascular events in patients with stable coronary heart disease: The heart and soul study. Archives of Internal Medicine. 2007;167(16):1798-803.

6. Kripalani S, Risser J, Gatti ME, Jacobson TA. Development and evaluation of the Adherence to Refills and Medications Scale (ARMS) among low-literacy patients with chronic disease. Value in health : the journal of the International Society for Pharmacoeconomics and Outcomes Research. 2009;12(1):118-23.

7. Matza LS, Park J, Coyne KS, Skinner EP, Malley KG, Wolever RQ. Derivation and validation of the ASK-12 adherence barrier survey. The Annals of pharmacotherapy. 2009;43(10):1621-30.

8. Hahn SR, Park J, Skinner EP, Yu-Isenberg KS, Weaver MB, Crawford B, et al. Development of the ASK-20 adherence barrier survey. Current medical research and opinion. 2008;24(7):2127-38.

9. Svarstad BL, Chewning BA, Sleath BL, Claesson C. The Brief Medication Questionnaire: a tool for screening patient adherence and barriers to adherence. Patient education and counseling. 1999;37(2):113-24.

10. Brooks CM, Richards JM, Kohler CL, Soong SJ, Martin B, Windsor RA, et al. Assessing adherence to asthma medication and inhaler regimens: a psychometric analysis of adult self-report scales. Medical care. 1994;32(3):298-307.

11. Choo PW, Rand CS, Inui TS, Lee ML, Cain E, Cordeiro-Breault M, et al. Validation of patient reports, automated pharmacy records, and pill counts with electronic monitoring of adherence to antihypertensive therapy. Medical care. 1999;37(9):846-57.

12. Kim MT, Hill MN, Bone LR, Levine DM. Development and testing of the Hill-Bone Compliance to High Blood Pressure Therapy Scale. Progress in cardiovascular nursing. 2000;15(3):90-6.

13. Chisholm MA, Lance CE, Williamson GM, Mulloy LL. Development and validation of the immunosuppressant therapy adherence instrument (ITAS). Patient education and counseling. 2005;59(1):13-20.

14. Clayton CD, Veach J, Macfadden W, Haskins J, Docherty JP, Lindenmayer JP. Assessment of clinician awareness of nonadherence using a new structured rating scale. Journal of psychiatric practice. 2010;16(3):164-9.

15. Morisky DE, Ang A, Krousel-Wood M, Ward HJ. Predictive validity of a medication adherence measure in an outpatient setting. Journal of clinical hypertension (Greenwich, Conn). 2008;10(5):348-54.

16. Reynolds K, Viswanathan HN, O'Malley CD, Muntner P, Harrison TN, Cheetham TC, et al. Psychometric properties of the Osteoporosis-specific Morisky Medication Adherence Scale in postmenopausal women with osteoporosis newly treated with bisphosphonates. The Annals of pharmacotherapy. 2012;46(5):659-70.

17. Lewis SJ, Abell N. Development and Evaluation of the Adherence Attitude Inventory. Research on Social Work Practice. 2002;12(1):107-23.

18. Morisky DE, Green LW, Levine DM. Concurrent and predictive validity of a self-reported measure of medication adherence. Medical care. 1986;24(1):67-74.

19. Ogedegbe G, Mancuso CA, Allegrante JP, Charlson ME. Development and evaluation of a medication adherence self-efficacy scale in hypertensive African-American patients. Journal of clinical epidemiology. 2003;56(6):520-9.

20. Fernandez S, Chaplin W, Schoenthaler AM, Ogedegbe G. Revision and validation of the medication adherence self-efficacy scale (MASES) in hypertensive African Americans. Journal of behavioral medicine. 2008;31(6):453-62.

21. Unni EJ, Farris KB. Development of a new scale to measure self-reported medication nonadherence. Research in social & administrative pharmacy : RSAP. 2015;11(3):e133-43.

22. Risser J, Jacobson TA, Kripalani S. Development and psychometric evaluation of the Self-efficacy for Appropriate Medication Use Scale (SEAMS) in low-literacy patients with chronic disease. Journal of nursing measurement. 2007;15(3):203-19.

23. Dolder CR, Lacro JP, Warren KA, Golshan S, Perkins DO, Jeste DV. Brief evaluation of medication influences and beliefs: development and testing of a brief scale for medication adherence. Journal of clinical psychopharmacology. 2004;24(4):404-9.

24. Lee S, Bae YH, Worley M, Law A. Validating the modified drug adherence Work-Up (M-DRAW) Tool to identify and address barriers to medication adherence. Pharmacy. 2017;5(3):52.

25. Grymonpre RE, Didur CD, Montgomery PR, Sitar DS. Pill count, self-report, and pharmacy claims data to measure medication adherence in the elderly. Annals of Pharmacotherapy. 1998;32(7-8):749-54.

26. Kerr T, Hogg RS, Yip B, Tyndall MW, Montaner J, Wood E. Validity of self-reported adherence among injection drug users. Journal of the International Association of Physicians in AIDS Care. 2008;7(4):157-9.

27. Willey C, Redding C, Stafford J, Garfield F, Geletko S, Flanigan T, et al. Stages of change for adherence with medication regimens for chronic disease: development and validation of a measure. Clinical therapeutics. 2000;22(7):858-71.

28. Godin G, Gagne C, Naccache H. Validation of a self-reported questionnaire assessing adherence to antiretroviral medication. AIDS patient care and STDs. 2003;17(7):325-32.

29. Hogan TP, Awad AG, Eastwood R. A self-report scale predictive of drug compliance in schizophrenics: reliability and discriminative validity. Psychological medicine. 1983;13(1):177-83.

30. Martinez CER, Sossa MP, Rand CS. Validation of a questionnaire for assessing adherence to metered-dose inhaler use in asthmatic children. Pediatric Asthma, Allergy & Immunology. 2007;20(4):243-54.

31. Schroeder K, Fahey T, Hay AD, Montgomery A, Peters TJ. Adherence to antihypertensive medication assessed by self-report was associated with electronic monitoring compliance. Journal of clinical epidemiology. 2006;59(6):650-1.

32. Bell DJ, Kapitao Y, Sikwese R, van Oosterhout JJ, Lalloo DG. Adherence to antiretroviral therapy in patients receiving free treatment from a government hospital in Blantyre, Malawi. Journal of acquired immune deficiency syndromes (1999). 2007;45(5):560-3.

33. Horne R, Weinman J, Hankins M. The beliefs about medicines questionnaire: The development and evaluation of a new method for assessing the cognitive representation of medication. Psychology & Health. 1999;14(1):1-24.

34. Greaves CJ, Hyland ME, Halpin DM, Blake S, Seamark D. Patterns of corticosteroid medication use: non-adherence can be effective in milder asthma. Primary care respiratory journal : journal of the General Practice Airways Group. 2005;14(2):99-105.

35. Dima AL, van Ganse E, Laforest L, Texier N, de Bruin M, group tA-L. Measuring medication adherence in asthma: Development of a novel self-report tool. Psychology & health. 2017;32(10):1288-307.

36. de Klerk E, van der Heijde D, Landewe R, van der Tempel H, van der Linden S. The compliance-questionnaire-rheumatology compared with electronic medication event monitoring: a validation study. The Journal of rheumatology. 2003;30(11):2469-75.

37. Wetzels G, Nelemans P, van Wijk B, Broers N, Schouten J, Prins M. Determinants of poor adherence in hypertensive patients: development and validation of the "Maastricht Utrecht Adherence in Hypertension (MUAH)-questionnaire". Patient education and counseling. 2006;64(1-3):151-8.

38. Kleppe M, Lacroix J, Ham J, Midden C. The development of the ProMAS: a Probabilistic Medication Adherence Scale. Patient preference and adherence. 2015;9:355-67.

39. Munoz-Moreno JA, Fumaz CR, Ferrer MJ, Tuldra A, Rovira T, Viladrich C, et al. Assessing self-reported adherence to HIV therapy by questionnaire: the SERAD (Self-Reported Adherence) Study. AIDS research and human retroviruses. 2007;23(10):1166-75.

40. Knobel H, Alonso J, Casado JL, Collazos J, Gonzalez J, Ruiz I, et al. Validation of a simplified medication adherence questionnaire in a large cohort of HIV-infected patients: the GEEMA Study. AIDS (London, England). 2002;16(4):605-13.

41. Jonsdottir H, Opjordsmoen S, Birkenaes AB, Engh JA, Ringen PA, Vaskinn A, et al. Medication adherence in outpatients with severe mental disorders: relation between self-reports and serum level. Journal of clinical psychopharmacology. 2010;30(2):169-75.

42. Fodor GJ, Kotrec M, Bacskai K, Dorner T, Lietava J, Sonkodi S, et al. Is interview a reliable method to verify the compliance with antihypertensive therapy? An international central-European study. Journal of hypertension. 2005;23(6):1261-6.

43. Shima R, Farizah H, Majid HA. The 11-item Medication Adherence Reasons Scale: reliability and factorial validity among patients with hypertension in Malaysian primary healthcare settings. Singapore medical journal. 2015;56(8):460.

44. Chung WW, Chua SS, Lai PSM, Morisky DE. The Malaysian Medication Adherence Scale (MALMAS): Concurrent Validity Using a Clinical Measure among People with Type 2 Diabetes in Malaysia. PLoS ONE. 2015;10(4):e0124275.

45. Najimi A, Mostafavi F, Sharifirad G, Golshiri P. Development and study of self-efficacy scale in medication adherence among Iranian patients with hypertension. Journal of education and health promotion. 2017;6.

46. George J, Mackinnon A, Kong DC, Stewart K. Development and validation of the Beliefs and Behaviour Questionnaire (BBQ). Patient education and counseling. 2006;64(1-3):50-60.

47. Thompson K, Kulkarni J, Sergejew AA. Reliability and validity of a new Medication Adherence Rating Scale (MARS) for the psychoses. Schizophr Res. 2000;42(3):241-7.

48. Lambert EV, Steyn K, Stender S, Everage N, Fourie JM, Hill M. Cross-cultural validation of the hill-bone compliance to high blood pressure therapy scale in a South African, primary healthcare setting. Ethnicity & disease. 2006;16(1):286-91.
