# Supplementary material for: Development of a PATIENT-Medication Adherence Instrument (P-MAI) and a HEALTHCARE PROFESSIONAL-Medication Adherence Instrument (H-MAI) using the nominal group technique
Source: PLoS One. 2020 Nov 11;15(11):e0242051. doi: 10.1371/journal.pone.0242051 (PMC7657514; doi:10.1371/journal.pone.0242051)
Supplement: S2 Table — (DOCX) [file pone.0242051.s002.docx]

**S2 Table:** The PATIENT-Medication Adherence Instrument (PMAI-9)

Please answer the following questions by placing a tick (√) in the appropriate box

| Section A: Adherence | | | | | | |
| --- | --- | --- | --- | --- | --- | --- |
|  | For the past 2 weeks.. | ^1^Strongly disagree | ^2^Disagree | ^3^Neutral | ^4^Agree | ^5^Strongly Agree |
| 1. | I do not take medication(s) MORE than directed |  |  |  |  |  |
| 2. | I do not take medication(s) LESS than directed |  |  |  |  |  |
| Section B: Knowledge and belief | | | | | | |
| 3. | I take my medication(s) everyday as directed |  |  |  |  |  |
| 4. | I have a good understanding of my illness |  |  |  |  |  |
| 5. | I am confident that my medication(s) are helping me |  |  |  |  |  |
| 6. | I am satisfied with the information that my doctor has shared with me |  |  |  |  |  |
| 7. | I am able to make a decision together with my doctor regarding the medication(s) that have been given to me |  |  |  |  |  |
| 8. | I know how to take my medication(s) (eg. dose, frequency) |  |  |  |  |  |
| 9. | I know why I am taking my medication(s) (eg. indication) |  |  |  |  |  |
