# Supplementary material for: Development of a PATIENT-Medication Adherence Instrument (P-MAI) and a HEALTHCARE PROFESSIONAL-Medication Adherence Instrument (H-MAI) using the nominal group technique
Source: PLoS One. 2020 Nov 11;15(11):e0242051. doi: 10.1371/journal.pone.0242051 (PMC7657514; doi:10.1371/journal.pone.0242051)
Supplement: S3 Table — (DOCX) [file pone.0242051.s003.docx]

**S3 Table:** The HEALTHCARE-PROFESSIONAL-Medication Adherence Instrument (H-MAI-9)

Please answer the following questions by placing a tick (√) in the appropriate box

| Section A: Adherence | | | | | | |
| --- | --- | --- | --- | --- | --- | --- |
|  |  | ^1^Strongly disagree | ^2^Disagree | ^3^Neutral | ^4^Agree | ^5^Strongly Agree |
| 1. | My patient does not take his/her medication(s) MORE than directed |  |  |  |  |  |
| 2. | My patient does not take his/her medication(s) LESS than directed |  |  |  |  |  |
| Section B: Knowledge and belief | | | | | | |
| 3. | My patient is taking his/her medication(s) everyday as directed |  |  |  |  |  |
| 4. | My patient has a good understanding of his/her illness |  |  |  |  |  |
| 5. | My patient is confident that his/her medication(s) are helping him/her |  |  |  |  |  |
| 6. | My patient is satisfied with the information shared by his/her doctor |  |  |  |  |  |
| 7. | My patient is able to make a decision together with his/her doctor regarding his/her medication(s) |  |  |  |  |  |
| 8. | My patient knows how to take his/her medication(s) (eg. dose, frequency) |  |  |  |  |  |
| 9. | My patient knows why he/she is taking their medication(s) (eg. indication) |  |  |  |  |  |
